# Study on Health Assessment Method of a Braking System of a Mine Hoist

**DOI:** 10.3390/s19040769

**Published:** 2019-02-13

**Authors:** Juanjuan Li, Guoying Meng, Guangming Xie, Aiming Wang, Jun Ding, Wei Zhang, Xingwei Wan

**Affiliations:** 1 School of Mechanical Electronic &Information Engineering, China University of Mining and Technology (Beijing), Beijing 100083, China; lantianljj@163.com (J.L.); wam_master@163.com (A.W.); dingjun0823@163.com (J.D.); 13326282690@163.com (X.W.); 2 College of Engineering, Peking University, Beijing 100871, China; xiegming@pku.edu.cn; 3 School of Mechanical-Electrical Engineering, North China Institute of Science and Technology, Yanjiao 101601, China; 4 Luoyang Zhongzhong automation engineering co., LTD, Luoyang 471039, China; 13663892507@163.com

**Keywords:** mine hoist, braking system, fuzzy comprehensive assessment (FCA), health assessment, neural network, health management

## Abstract

This paper presents a method for calculating the health degree (HD) of a braking system of a mine hoist combined with three-level fuzzy comprehensive assessment (TLFCA) and a back-propagation neural network (BPNN). Firstly, the monitored values of a sensor are fused by multi-time fusion and the fuzzy comprehensive assessment values (FCAVs) of the health condition (HC) of the sensor are obtained. Secondly, the FCAVs of all sensors in a subsystem are fused by multi-sensor fusion, and FCAVs of the subsystem are obtained. Then the FCAVs of all subsystems are fused by multi-subsystem fusion and FCAVs of the system are obtained. All the FCAVs are fed into a pre-trained neural network, and the corresponding HD of the sensor, subsystem and system is obtained. Finally, the practicability, reliability and sensitivity of the proposed method are verified by the monitored values of the test rig. This paper presents a method to provide technical support for intelligent maintenance, and also provides necessary data for further prognostics health management (PHM) of the braking system. The method presented in this paper can also be used as a reference for the HD calculation of the whole hoist and other complicated equipment.

## 1. Introduction

As a key equipment in the mine production, the mine hoist constructs the connection between ground level and the underground, and shoulders the important task of lifting coal, ore, personnel, materials and equipment. With the rapid development of science and technology, the mine hoist is developing towards the large scale, complication, automation and intelligence. The braking system is the last safeguard to ensure the safe operation of the hoist. Once the braking system fails, it may affect the hoist or even cause the whole mine to shut down. A survey of hoist accidents finds that the accidents caused by braking system faults account for more than 60% of all hoist accidents. Therefore, it is imperative to improve the safety and reliability of the hoist braking system.

Prognostics health management (PHM) is proposed to meet the requirement of self-protection and independent diagnosis, and it is the upgrade and development of condition-based maintenance (CBM). It emphasizes the condition perception in the equipment management, monitors the equipment health condition (HC), and the frequent fault area and period, and predicts fault occurrence through the data monitoring and analysis, thus greatly improving the reliability and operational maintenance efficiency.

PHM was proposed by the U.S. Department of Energy and the U.S. Department of Defense in the development of army-equipped helicopters [1,2]. The theory was developed and widely used in the health management of aerospace [3,4], naval ship [5], wind turbine [6], power plant [7], rotating machinery [8,9], electronic equipment [10] and other large and sophisticated equipment, which provides a reliable guarantee for safe operation. As one of the core technologies of PHM, equipment health assessment has been extensively studied [11] and widely used in liquid hydrogen supply systems [12], aircraft hydraulic systems [13], aeroengines [14], wind tunnel equipment [15], and so on. Because of the importance of hoists in mine production, many scholars have done research on the condition monitoring [16,17,18] and fault diagnosis [19,20,21] of hoist activity, and achieved many results [22,23]. However, the health assessment of the hoist and braking systems is seldom studied. At present, the implemented standards of health assessment of hoists are all issued by authoritative organizations, such as the national standard, the industry standard, the international standard, and so on, e.g. coal mine safety regulations stipulate the following requirements for the braking system: (1) the safety braking deceleration must be less than or equal to 5 m/s^2^ during the vessel’s ascent with a heavy load, and must be greater than or equal to 1.5 m/s^2^ during the vessel’s descending with heavy-load in vertical well. (2) The idle time of disc brake, which refers to the time from the closing of the protection circuit to the contact time between the brake shoe and the brake disc, should not exceed 0.3 s. (3) The gap between brake shoe and brake disc should not be greater than 2mm, generally between 0.5–1.5 mm. These assessment standards, which can be called threshold or limit assessment standards, are all for a single parameter and lack of health assessment of the system and the overall hoist. In this paper, a method of assessing the health degree (HD) of a mine hoist braking system based on the combination of three-level fuzzy comprehensive assessment (TLFCA) and back-propagation neural network (BPNN) is proposed.

The rest of this paper is structured as follows: Section 1 introduces the method of calculating the HD of a mine hoist based on the combination of TLFCA and BPNN. Section 2 introduces the fuzzy comprehensive assessment (FCA) method. Section 3 first introduces the hoist and braking system, and then introduces the TLFCA method and steps. Section 4 introduces the method of training a neural network by fuzzy comprehensive assessment values (FCAVs) and corresponding HD. Section 5 verifies the reliability, stability and sensitivity of the proposed method by examples. Section 6 summarizes the advantages and disadvantages of the method and its further development and application.

## 2. Method of Calculating Health Degree (HD) Based on Combination of Three-Level Fuzzy Comprehensive Assessment (TLFCA) and Back-Propagation Neural Network (BPNN)

**Figure 1:**
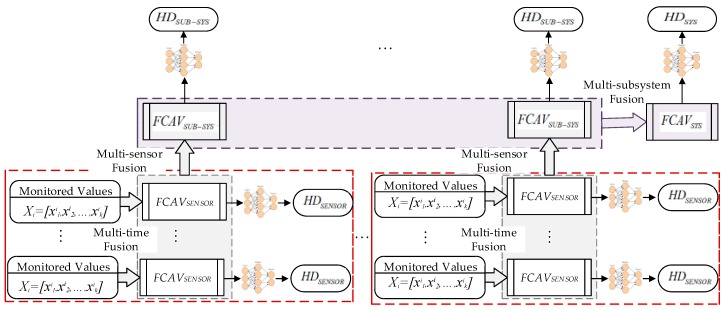
Block diagram of method of calculating health degree (HD) based on the combination of three-level fuzzy comprehensive assessment (TLFCA) and back-propagation neural network (BPNN).

Since Professor Zadeh, a cybernetics expert in the United States, put forward the concept of fuzzy sets, fuzzy mathematics has developed rapidly in recent decades, and FCA based on fuzzy mathematics theory has also been studied and widely applied [2,24,25,26,27]. The basic idea of FCA is to make a reasonable comprehensive assessment through considering the factors related to the assessed affairs by using the principle of fuzzy linear transformation and maximum membership degree. The FCA method provides a new mathematical tool for solving multi-objective assessment and decision problem under fuzzy environment. As a composite system with several subsystems, there are many factors that need to be considered in the health assessment of hoist braking system. With the characteristic of “fuzziness”, it is difficult to measure quantitatively the influence that the factors have on each other and on the HC of the system. Therefore, it is suitable to use the method of FCA to assess the HC of the braking system of the mine hoist. However, the principle of maximum membership ignores the contribution of non-maximum membership to HD, and the result is only a certain HC, not the degree belonging to the HC. HD is a number within the scope of [0,1], representing the health value of an assessed object. This method not only makes the assessment result express the degree belonging to a certain HC accurately, but also accords with people’s thinking habits. In this paper, a method for assessing the HD of mine hoist breaking system based on the combination of TLFCA and BPNN is proposed. The block diagram of the method is shown in Figure 1. Firstly, the data fusion of multiple time points is carried out by the sensor output samples, and the FCAVs of a sensor are obtained. Then, the FCAVs of multiple sensors in each subsystem are fused in space, and the FCAVs of the subsystem are obtained. Finally, the BPNN is used to accomplish the defuzzification calculation of HD from the FCAVs.

## 3. Introduction to the Fuzzy Comprehensive Assessment (FCA) Method

The basic idea of fuzzy assessment is using the principle of fuzzy transformation and maximum membership degree. In order to make a reasonable comprehensive assessment of the assessment object, we should consider all the factors related to the assessed factors, standardize each index, and distribute the weight according to the influence of the different index. In the comprehensive assessment of a complex system, because there are many factors, and each factor must be given a certain weight, it is likely that the following situation will occur: ① Difficulty in determining appropriate weight; ② Lack of meaningful assessment results, etc. For such a problem, the method of multi-level FCA can be used, which means the factors are divided into several layers according to the characteristics, and the FCA of each type at the lowest level is carried out firstly to obtain the assessment matrix of the upper layer. Then the FCA of each class at the upper layer is carried out, and the assessment matrix at the upper layer can be obtained. In a similar fashion, the final result of the problem can be obtained. The steps of the two-level FCA are as follows, and the multi-level assessment method continues to be subdivided on the basis of the two-level.

Step 1: Determine the factor sets of the assessed object, denoted by U:

(1) $$\left\{ \begin{array}{l} U=\left[U_{1},U_{2},\cdots ,U_{s}\right]\\ U_{i}=\left[U_{i1},U_{i2},\cdots ,U_{ik_{i}}\right]\\ s.t.\;\sum_{i=1}^{s}k_{i}=n\\ s.t.\;\left(\forall i,j\right)\left(i\neq j\to U_{i}\cap U_{j}=\emptyset \right) \end{array}\right.$$

where $U_{i}$ is the *i*th factor class, $U_{ij}$ is the the *j*th factor in *i*th factor class, *s* is the number of factor classes, $k_{i}$ is the number of factors in the *i*th factor class, and *n* is the total number of factors.

Step 2: Determination assessment sets, denoted by V:

(2) $$V=\left[V_{1},V_{2},\cdots ,V_{m}\right]$$

where $V_{l}$ is the *l*th assessment factor, and *m* is the number of assessment factors.

Step 3: Determine the weighting sets

(1) The weight sets of the factor sets, denoted by W:

(3) $$\left\{ \begin{array}{l} W=\left[W_{1},W_{2},\cdots ,W_{s}\right]\\ s.t.\;\sum_{i=1}^{s}W_{i}=1 \end{array}\right.$$

where $W_{i}$ is the weight of $U_{i}$.

(2) The weight sets of factor classes, denoted by $\omega_{i}$:

(4) $$\left\{ \begin{array}{l} \omega_{i}=\left[\omega_{i1},\omega_{i2},\cdots ,\omega_{ik_{i}}\right]\\ s.t.\;\sum_{j=1}^{k_{i}}\omega_{ij}=1 \end{array}\right.i=1,2,\cdots ,s$$

where $\omega_{ij}$ is the weight of $U_{ij}$.

Step 4: Determine the fuzzy conversion matrix between $U_{i}$ and $V_{l}$ denoted by $R_{i}$:

(5) $$R_{i}=\left[\begin{array}{cccc} R_{11}^{i} & R_{12}^{i} & \cdots & R_{1m}^{i}\\ R_{21}^{i} & R_{22}^{i} & \cdots & R_{2m}^{i}\\ \vdots & \vdots & \vdots & \vdots \\ R_{k_{i}1}^{i} & R_{k_{i}2}^{i} & \cdots & R_{k_{i}m}^{i} \end{array}\right]$$

where $R_{jl}^{i}$ is fuzzy conversion value of the *j*th factor in the *i*th factor class and the *l*th assessment factors.

Step 5: Calculate the FCAVs of the *i*th factor class by fuzzy matrix compound operation, denoted by $FCAV_{i}$:

(6) $$FCAV_{i}=\omega_{i}\circ R_{i}=\left(FCAV_{i1},FCAV_{i2},\cdots ,FCAV_{im}\right),\;i=1,2,\cdots ,s$$

where ∘ is fuzzy operator, $FCAV_{il}=\begin{array}{cc} \sum_{j=1}^{k_{i}}\left(\omega_{ij}\cdot R_{jl}^{i}\right), & l=1,2,\cdots ,m \end{array}$, and $FCAV_{il}$ is the FCAV of the *l*th assessment factor in the *i*th factor class. 

Step 6: Determine the fuzzy relationship matrix between U and V, denoted by R:

(7) $$R=\left[\begin{array}{c} FCAV_{1}\\ FCAV_{2}\\ \vdots \\ FCAV_{s} \end{array}\right]=\left[\begin{array}{cccc} FCAV_{11} & FCAV_{12} & \cdots & FCAV_{1m}\\ FCAV_{21} & FCAV_{22} & \cdots & FCAV_{2m}\\ \vdots & \vdots & \vdots & \vdots \\ FCAV_{s1} & FCAV_{s2} & \cdots & FCAV_{sm} \end{array}\right]$$

Step 7: Obtained FCAVs of the factor sets by fuzzy matrix compound operation, denoted by FCAV:

(8) $$FCAV=W\circ R=\left(FCAV^{1},FCAV^{2},\cdots ,FCAV^{m},\right)$$

where ∘ is fuzzy operator, $FCAV^{1}$ is the FCAV of the factor sets belongs to the *l*th assessment factor.

## 4. Comprehensive Assessment of the Health Condition (HC) of Braking System

### 4.1. Introduction of Hoist and Braking System

**Figure 2:**
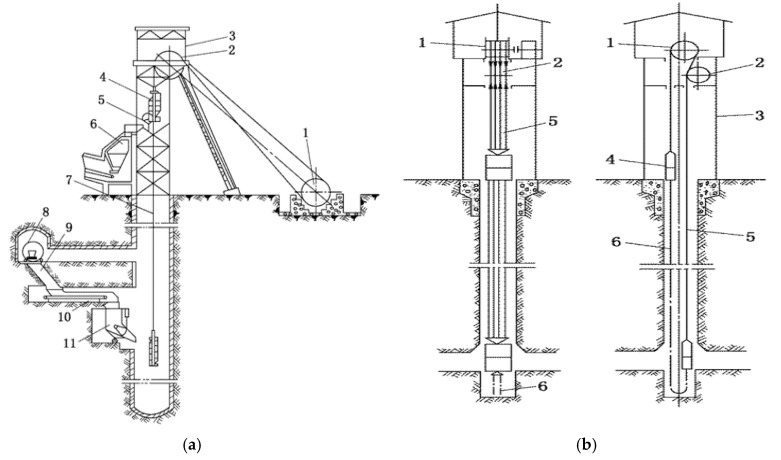
Schematic diagram of mine hoist. (**a**) single rope winding hoist. 1—hoisting pulley; 2—hoisting sheave; 3—derrick; 4—skip; 5—dump track; 6—ground coal bunker; 7—wire rope; 8—dumper; 9—underground coal bin; 10—conveyer; 11—ration bucket. (**b**) tower-type multi-rope friction hoist. 1—driving wheel; 2—guide wheel; 3—shaft tower; 4—skip; 5—wire rope; 6—tail rope.

A hoist mainly consists of hoisting container, hoisting rope, hoisting device, derrick, hoisting sheave, loading and unloading equipment and accessories. The mine hoists used in China are mainly single-rope winding hoists and multi-rope friction hoists. Their schematic diagrams are shown in Figure 2a,b respectively.

**Figure 3:**
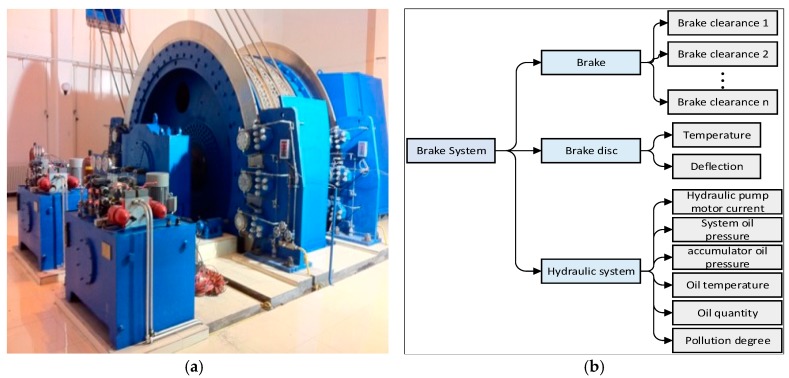
(**a**) Scene photograph of braking system. (**b**) Block diagram of sensor installation in braking system.

The brake system of hoist consists of brake, hydraulic transmission system and control system, with the functions of working brake, stopping brake, safety brake, and regulation rope brake. Brake clearance (BC), brake disc deflection, brake disc temperature, the motor current of tjhe hydraulic pump, oil pressure of system, oil temperature, liquid level and oil pressure of accumulator are installed in the braking system to monitor the HC of braking system in real time [28,29,30,31,32]. The scene photograph of braking system is shown in Figure 3a, and the block diagram of sensor installation in the braking system is shown in Figure 3b:

### 4.2. Setting Up Factor Sets

According to the block diagram of sensor installation in braking system, the monitored parameters of brake clearance, brake disk deflection, brake disc temperature, motor current of hydraulic pump, oil pressure of system, oil temperature, liquid level and oil pressure of accumulator are chosen as the assessment factor sets. The BCs of all brakes constitute the brake subsystem. Brake disc deflection and brake disc temperature constitute the brake disc subsystem. The hydraulic subsystem is constituted by motor current of hydraulic pump, oil pressure of system, oil temperature, oil quantity, contamination degree and oil pressure of the accumulator. That is, the factor sets of the braking system:

$U=\left[U_{1},U_{2},U_{3}\right]=\;[\mathrm{brake},\;\mathrm{brake}\;\mathrm{disc},\;\mathrm{hydraulic}\;\mathrm{system}]$

$U_{1}=\left[U_{11},U_{12},\cdots ,U_{18}\right]=\left[BC_{1},BC_{2},\cdots ,BC_{8}\right]$

$U_{2}=\left[U_{21},U_{22}\right]=\;[\mathrm{temperature},\;\mathrm{deflection}]$

$\begin{array}{ll} U_{3} & =\left[U_{31},U_{32},U_{33},U_{34},U_{35}\right]\\ & =[\mathrm{motor}\;\mathrm{current}\;\mathrm{of}\;\mathrm{hydraulic}\;\mathrm{pump},\;\mathrm{oil}\;\mathrm{pressure}\;\mathrm{of}\;\mathrm{system}\;,\;\mathrm{oil}\;\mathrm{temperature},\\ & \;\mathrm{oil}\;\mathrm{quantity},\;\mathrm{pollution}\;\mathrm{degree},\;\mathrm{oil}\;\mathrm{pressure}\;\mathrm{of}\;\mathrm{accumulator}] \end{array}$

### 4.3. Setting Up Assessment Sets

Based on the actual needs of the evaluation decision, and the degradation degree of the actual operation, four different categories of HC are defined, namely health state (HS), sub-health (SH), critical fault (CF), and fault state (FS). Four categories of HC can be described as follows:

HS: The whole braking system or the subsystems such as brake, brake disc and hydraulic system is very healthy. All sensor parameters are also healthy, and their measurement data are close to expectations.

SH: The whole braking system or the subsystems such as brake, brake disc and hydraulic system is in SH, which is a state between HS and CF. All sensor parameters data may fluctuate near their expectations, but within the normal range. In general, most HC is between HS and SH.

CF: The braking system or the subsystems such as brake, brake disc and hydraulic system is in the CF, which is a transition state. Their actual measurements have deviated from expectations relative to the SH state, but not completely.

FS: The braking system or the subsystems such as brake, brake disc and hydraulic system is in FS. The actual outputs of most sensors or sensors with high importance are completely different from its expected results.

Four different categories of HC are considered as criteria for health assessment, that is, the assessment sets for HC:

$V=\left[V_{1},V_{2},V_{3},V_{4}\right]=\left[HS,SH,CF,FS\right]$.

### 4.4. Standardized Processing of the Monitored Values

The monitored values have different physical significances and ranges. In order to carry on the comprehensive analysis, standardized processing is required, that is, the monitored data should be converted to [0,1]. According to the influence of monitored values to equipment performance, the monitored values can be divided into three types: benefit type, cost type and interval type. The greater the value of the benefit type, the better the performance of the equipment will be, and the smaller the value of the cost type, the better the performance of the equipment. Combining the characteristics of benefit and cost, when the interval type, value is in a certain range, the performance of the equipment is the best. The farther away from the range the value is, the worse the performance of the equipment will be. The standardized processing formula for each type datum is as follows:

(1): Benefit type

(9) $$g\left(x\right)=\left\{ \begin{array}{ll} 0 & x<x_{\min1}\\ \frac{x-x_{\min1}}{x_{\min2}-x_{\min1}} & x_{\min1}\leq x\leq x_{\min2}\\ 1 & x>x_{\min2} \end{array}\right.$$

(2): Cost type

(10) $$g\left(x\right)=\left\{ \begin{array}{ll} 1 & x<x_{\max2}\\ \frac{x_{\max1}-x}{x_{\max1}-x_{\max2}} & x_{\max2}\leq x\leq x_{\max1}\\ 0 & x>x_{\max1} \end{array}\right.$$

(3): Interval type

(11) $$g\left(x\right)=\left\{ \begin{array}{ll} 0 & x<x_{\min1}\\ \frac{x-x_{\min1}}{x_{\min2}-x_{\min1}} & x_{\min1}\leq x\leq x_{\min2}\\ 1 & x_{\min2}<x<x_{\max2}\\ \frac{x_{\max1}-x}{x_{\max1}-x_{\max2}} & x_{\max2}\leq x\leq x_{\max1}\\ 0 & x>x_{\max1} \end{array}\right.$$

where *x* is the monitored value, $\left[x_{\min1},x_{\max1}\right]$ is the required range of the lowest operation of the data, $\left[x_{\min2},x_{\max2}\right]$ is the required range of the best operation of the data, and $x_{\min1}\leq x_{\min2}<x_{\max2}\leq x_{\max1}$.

**Figure 4:**
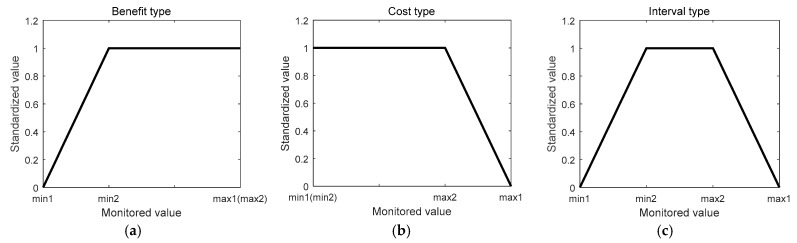
The standardized data. (**a**) Benefit type, (**b**) cost type, (**c**) interval type.

The standardized data are shown in Figure 4:

### 4.5. Determination of Fuzzy Membership Function

**Figure 5:**
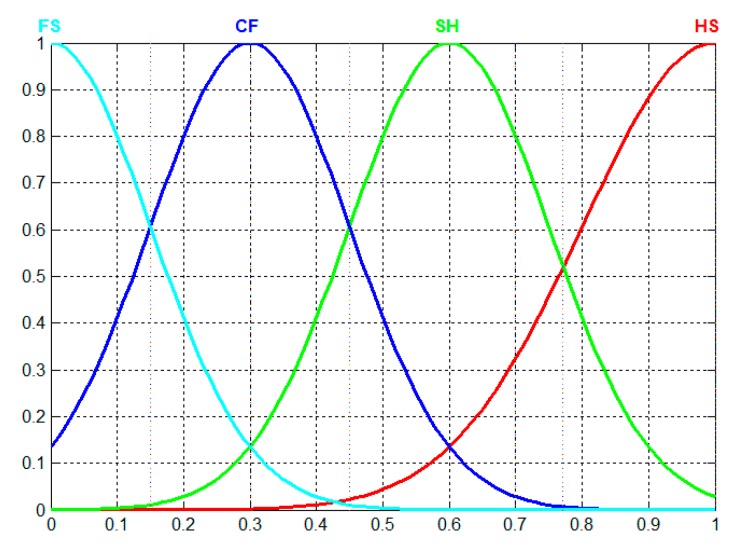
Membership functions of fuzzy sets.

The thought of membership function degree is the basic idea of fuzzy mathematics, and the key of applying fuzzy mathematics is to establish a membership function that accords with the reality. According to the analysis of the historical data of the acquired values of the braking system, it can be seen that during the steady-state operation, the acquired values obey the normal distribution, that is, the closer to the best estimation, the greater the probability of occurrence. For the HD of the braking system, the closer to the best estimation, the better the HC, so we choose the normal distribution function as the membership function of the fuzzy set of four categories of HC. Among them, the partial large and partial small normal distribution function is used for HS and FS, respectively, and the intermediate normal distribution function is used for SH and critical CF as shown in Figure 5. The membership functions of the four fuzzy subsets can be written as Formula 12:

(12) $$f_{V_{l}}\left(x\right)=\exp\left[-\frac{{\left\|x-\mu_{l}\right\|}^{2}}{2\delta_{l}^{2}}\right]$$

where $V_{l}$ represents various HC, l=1,2,3,4 respectively. $V_{1},V_{2},V_{3},V_{4}$ represent the four states of HS, SH, CF and FS. $\mu_{l}$ is the expected value of the *l*th HC. $\delta_{l}$ is the standard deviation of the *l*th HC. Reference paper [13] for $\mu_{l}$ and $\delta_{l}$ values, given according to the empirical method, the values are shown in Table 1.

### 4.6. Weight Vector Calculation

Determining the weight of each index reasonably is an important work to obtain reliable assessment results. There are many methods for weight determination, including subjective weight method, objective weight determination method and subjective and objective weight determination method. Subjective weight determination includes expert estimation, and an analytic hierarchy process (AHP). Objective weight determination, which is based on the inherent information contained in the assessment index, includes the weight determination method based on fuzzy distance, index variance and coefficient of variation, etc. In order to ensure the objectivity, impartiality and scientificalness of weight coefficient, this paper puts forward a method of weight determination which combines expert scoring, objective weight determination and AHP.

#### 4.6.1. Weight Sets of Sensors

(1) Experts score to determine the weight of each time point: $w_{i}^{1}=\left[w_{i1}^{1},w_{i2}^{1},\cdots ,w_{ik}^{1}\right]$, *k* is the number of time points.

(2) According to the characteristics of health management, the further the monitored value from the expected value, the greater the probability of fault is and the greater the weight is. So define the scale value of the *i*th parameter at the *j*th time point as Formula 13:

(13) $$d_{ij}=\frac{c_{ij}}{s_{ij}}\;\left(j=1,2,\cdots ,k\right)$$

where $c_{ij}$ is the absolute value of the difference between the monitored and expected value of the *i*th parameter at the *j*th point; $s_{ij}$ is the absolute value of the difference between the expected value of the health and fault of the *i*th parameter.

According to the Formula 13, we can get the scale value of the *i*th parameter at *j*th point: $d_{ij}=\left[d_{i1},d_{i2},\cdots ,d_{ik}\right]$, and then get the weight vector $w_{i}^{2}=\left[w_{i1}^{2},w_{i2}^{2},\cdots ,w_{ik}^{2}\right]$ by normalized scale value:

(14) $$w_{ij}^{2}=\frac{d_{ij}}{\sum_{j=1}^{k}d_{ij}}$$

(3) Determine the weight sets of the *i*th sensor $w_{i}$: After getting $w_{i}^{1}$ and $w_{i}^{2}$, the weight sets of the *i*th sensor are calculated as follows:

(15) $$w_{i}=\left[\frac{w_{i1}^{1}+w_{i1}^{2}}{\sum_{j=1}^{k}\left(w_{ij}^{1}+w_{ij}^{2}\right)},\frac{w_{i2}^{1}+w_{i2}^{2}}{\sum_{j=1}^{k}\left(w_{ij}^{1}+w_{ij}^{2}\right)},\cdots ,\frac{w_{ik}^{1}+w_{ik}^{2}}{\sum_{j=1}^{k}\left(w_{ij}^{1}+w_{ij}^{2}\right)}\right]=\left[w_{i1},w_{i2},\cdots ,w_{ik}\right]$$

#### 4.6.2. Weight Sets of Subsystems

(1) According to the FCAVs of each sensor or subsystem and the principle of maximum membership, if there is a parameter in FS, the weight of the parameter is 1 and the rest is 0.

(2) Elsewhere, if there is a parameter or subsystem in CF, the weight of each parameter or subsystem is determined by the proportion of the CF value in the sum of the CF, that is:

(16) $$\omega_{i}=\frac{FCAV_{CF}^{i}}{\sum_{i=1}^{n}FCAV_{CF}^{i}}$$

where $FCAV_{CF}^{i}$ is FCAV of the *i*th sensor or subsystem is in CF.

(3) If all HC of the sensors or subsystems is HS or SH, the weight of each sensor or subsystem is determined by AHP method. The AHP method can better quantify experts’ judgment of weight importance, and overcome the characteristics of weight arbitrariness.

### 4.7. Fuzzy Comprehensive Assessment Values (FCAVs) Calculation of a Sensor

**Figure 6:**
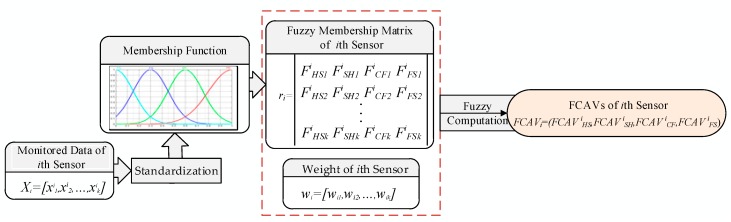
Flow chart of fuzzy comprehensive assessment value (FCAV) calculation for single parameter.

FCAVs of a sensor are calculated by fusing monitored values of multiple time points. The flow chart is shown in Figure 6. The steps are as follows:

Step 1: Standardize the monitored values.

Step 2: Plugging the standardized data into four membership functions respectively, we can obtain the fuzzy assessment values of each HC, and use these values to form the HC assessment matrix of the sensor.

(17) $$r_{i}=\left[\begin{array}{cccc} F_{HS1}^{i} & F_{SH1}^{i} & F_{CF1}^{i} & F_{FS1}^{i}\\ F_{HS2}^{i} & F_{SH2}^{i} & F_{CF2}^{i} & F_{FS2}^{i}\\ \vdots & \vdots & \vdots & \vdots \\ F_{HSk}^{i} & F_{SHk}^{i} & F_{CFk}^{i} & F_{FSk}^{i} \end{array}\right]$$

where $F_{HSn}^{i}$ is the FCAV of the *i*th sensor at the *n*th point is in HS; $F_{SHn}^{i}$ is the FCAV of the *i*th sensor at the *n*th point is in SH; $F_{CFn}^{i}$ is the FCAV of the *i*th sensor at the *n*th point is in CF; $F_{FSn}^{i}$ is the FCAV of the *i*th sensor at the *n*th point is in FS.

Step 3: Calculation of fuzzy comprehensive:

(18) $$\begin{array}{ll} FCAV_{i}=w_{i}\circ r_{i} & =[w_{i1},w_{i2},\cdots ,w_{ik}]\circ \left[\begin{array}{cccc} F_{HS1}^{i} & F_{SH1}^{i} & F_{CF1}^{i} & F_{FS1}^{i}\\ F_{HS2}^{i} & F_{SH2}^{i} & F_{CF2}^{i} & F_{FS2}^{i}\\ \vdots & \vdots & \vdots & \vdots \\ F_{HSk}^{i} & F_{SHk}^{i} & F_{CFk}^{i} & F_{FSk}^{i} \end{array}\right]\\ & =\left(\sum_{j=1}^{k}w_{j}^{i}*F_{HSj}^{i},\sum_{j=1}^{k}w_{j}^{i}*F_{SHj}^{i},\sum_{j=1}^{k}w_{j}^{i}*F_{CFj}^{i},\sum_{j=1}^{k}w_{j}^{i}*F_{FSj}^{i}\right)=\left(FCAV_{HS}^{i},FCAV_{SH}^{i},FCAV_{CF}^{i},FCAV_{SF}^{i}\right) \end{array}$$

### 4.8. FCAVs Calculation of Subsystem

**Figure 7:**
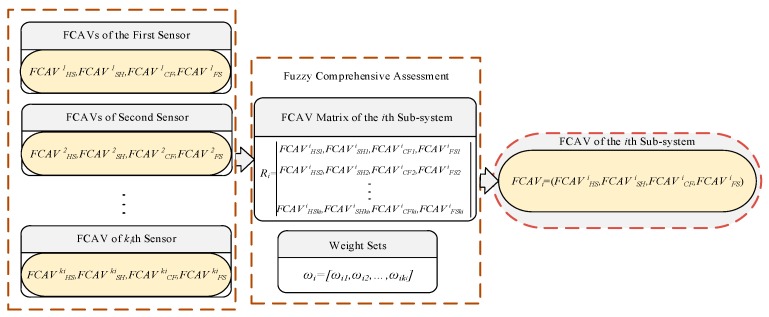
Flow chart of FCAVs calculation of subsystem.

The FCAVs of the subsystem are obtained through the fusion of the multi-sensor FCAVs, the flow chart is shown in Figure 7, and the steps are as follows:

Step 1: Composing subsystem assessment matrix by the FCAVs of each sensor:

(19) $$R_{i}=\left[\begin{array}{c} FCAV_{1}^{i}\\ FCAV_{2}^{i}\\ \vdots \\ FCAV_{k_{i}}^{i} \end{array}\right]=\left[\begin{array}{c} FCAV_{HS1}^{i},FCAV_{SH1}^{i},FCAV_{CF1}^{i},FCAV_{SF1}^{i}\\ FCAV_{HS2}^{i},FCAV_{SH2}^{i},FCAV_{CF2}^{i},FCAV_{SF2}^{i}\\ \vdots \\ FCAV_{HSk_{i}}^{i},FCAV_{SHk_{i}}^{i},FCAV_{CFk_{i}}^{i},FCAV_{SFk_{i}}^{i} \end{array}\right]$$

Step 2: Calculation of fuzzy comprehensive

(20) $$FCAV_{i}=\omega_{i}\circ R_{i}$$

### 4.9. FCAVs Calculation of Braking System

After obtaining the FCAVs of the brakes, brake discs and oil supply subsystems, the calculation method of the FCAVs of the braking system is the same as in Section 4.8.

## 5. Neural Network Training of HD Calculation

Fuzzyfication is the process of mapping the crisp values into the n-tuplets of membership functions degrees. The defuzzyfication is the transformation of the given membership function into a crisp value.

**Figure 8:**
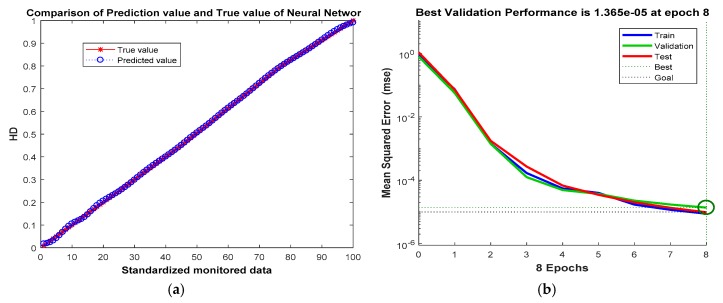
The performance test results of BPNN model. (**a**) The comparison between the predicted value and the true value; (**b**) The performance of BPNN model.

In this paper, BPNN is used to complete the calculation from FCAVs to HD. The specific method is taking the FCAVs obtained by FCA as input and calculating the value of HD by the trained neural network. The training of the neural network method is as follows:

In Figure 5, the standardized *x* is divided into 200 parts by step size 0.005, getting the fuzzy membership value and its HD value of four categories of HC corresponding to each *x* value, in which the 1~30 samples represent FS state, and their HD values are 0–0.15; 31–90 represent CF state, the corresponding HD values are 0.155–0.45; 91–145 represent SH state, the corresponding HD values are 0.455–0.8; 146–200 represent HS state, the corresponding HD values are 0.805–1. Using the odd samples trains BPNN to include 4 input neurons, 9 hidden layer neurons, and 1 output neuron [33], Sigmoid transmission function, minimum error of 0.00001 and learning rate of 0.05 are chosen. The performance of the BPNN model is tested with the even samples, as shown in Figure 8.

## 6. Example Calculation

### 6.1. Introduce Test Bed

**Figure 9:**
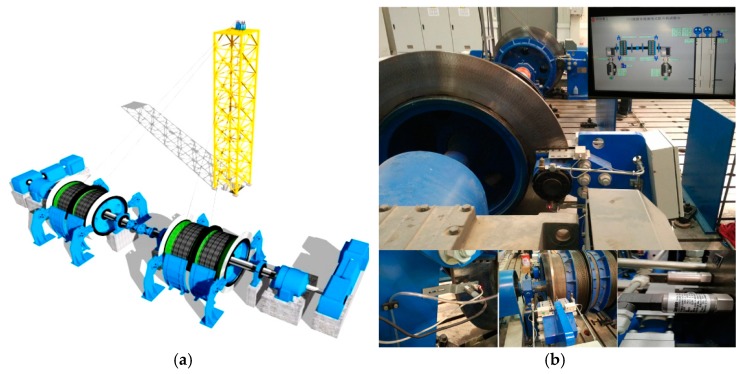
The test-bed. (**a**) The design drawing; (**b**) the site photos.

In this paper, the test bed is the test platform system of the ultra-deep well hoist of CITIC Heavy Industries. According to the similarity theory, the test bed is reduced to 0.1 of the actual hoist, and the main parameters of the test bed are shown in Table 2. Sensors of BC, brake disc temperature, deflection and so on are installed on the test-bed. The design drawing and site photos are shown in Figure 9.

### 6.2. Sensor Data Acquisition

The on-line monitored values of the test bench is used to verify the health assessment method proposed in this paper. The sampling frequency of each sensor in the test bed is set to 10 Hz, and in the data fusion of multiple time points, the number of sampling point is set to 8. A set of on-line monitored values is shown in Table 3 and T-1 to T-8 are the measured value of 8 time points. These measured values should be standardized with Formulas 9, 10 or 11 before being brought into Formula 12.

### 6.3. Calculation of FCAVs of a Sensor

Because the calculation process of each sensor is the same, for the convenience of narration, the BC 1-1 sensor is now used as an example to explain the HD calculation process:

Step 1: The standardized value is brought into Formula 12, and the assessment matrix is obtained based on Formula 17:

$$r_{1}=\left[\begin{array}{cccc} F_{HS1}^{1} & F_{SH1}^{1} & F_{CF1}^{1} & F_{FS1}^{1}\\ F_{HS2}^{2} & F_{SH2}^{2} & F_{CF2}^{2} & F_{FS2}^{2}\\ \vdots & \vdots & \vdots & \vdots \\ F_{HSk}^{8} & F_{SHk}^{8} & F_{CFk}^{8} & F_{FSk}^{8} \end{array}\right]=\left[\begin{array}{llll} 0.4578 & 0.6065 & 0.0111 & 0\\ 0.4868 & 0.5662 & 0.0091 & 0\\ 0.2163 & 0.946 & 0.0657 & 0.0001\\ 0.1353 & 1 & 0.1353 & 0.0003\\ 0.2163 & 0.946 & 0.0657 & 0.0001\\ 0.3247 & 0.8007 & 0.0286 & 0\\ 0.3753 & 0.7261 & 0.0198 & 0\\ 0.5461 & 0.4868 & 0.006 & 0 \end{array}\right]$$

Step 2: Use expert scoring to determine weights: Step 2: Use expert scoring to determine weights: 

$w_{1}^{1}=\left[0.125,0.125,0.125,0.125,0.125,0.125,0.125,0.125\right]$

Step 3: Get weights based on Formula 14:

$w_{1}^{2}=[0.1046\;0.1004\;0.1464\;0.1674\;0.1464\;0.1255\;0.1172\;0.0921]$

Step 4: Get comprehensive weights based on Formula 15:

$w_{1}=[0.1075\;0.1039\;0.1434\;0.1613\;0.1434\;0.1254\;0.1183\;0.0968]$

Step 5: Get FCAVs based on Formula 18:

$FCAV_{BC1-1}=[0.3216\;0.7900\;0.0493\;0.0001]$

Step 6: Use neural network to calculate the HD of BC 1-1:

$HD_{BC1-1}=0.7219$

From the calculation results, it can be seen that the HD value of BC 1-1 is 0.7219, the HC is in SH, so the braking system can continue to run.

Table 4 show the FCAVs, HD and HC of the sensors in brake system.

It can be seen from Table 4 that the data of the BC sensors 2-1, 2-2 and 3-1 are close to the expected value, with the corresponding HD 0.8156, 0.8146 and 0.8259 respectively; the BC sensor 4-2 data is slightly farther to the expected value, and the corresponding HD is 0.668; the BC 3-2 data is farther away from the expectation, and its HD is 0.2502; the BC 4-1 data is the furthest from the expectation, and many measurements have exceeded the bounds that can be allowed to run, so its HD is 0.1371. The verification results tally with the actual situation, the closer the fault, the lower the HD value, and the sensors of the brake disc and hydraulic subsystem also tally with the actual situation.

### 6.4. Calculation of FCAVs of System and Subsystem

According to the method introduced in Section 4.8 and Section 4.9, the FCAVs, HD and HC of subsystems and brake system are listed in Table 5.

As shown in the above Tables: due to the failure of BC 4-1, in the absence of other sensors failure, the brake subsystem and the whole brake system are in FS, which tallies with the actual situation, indicating the practicality of the method proposed in this paper.

In order to study the sensitivity of the method proposed in this paper, assuming that the values monitored by BC 4-1 in Table 3 are changed and other sensor-monitored values remain unchanged, the HD of subsystems and system are studied. The four sets of data in Table 6 are monitored by BC 4-1 in HS, SH, CF and FS respectively. The HD and HC of BC 4-1, brake subsystem and braking system calculated by different data are also shown in Table 6.

From Table 6 below, we can see that as the BC 4-1 data changes from near expectations to away from expectations slowly, the HD of the sensor changes from 0.9803 to 0.1371, the HD of the brake subsystem changes from 0.451 to 0.1371, and the HD of the system changes from 0.6944 to 0.1371. The reason for the low HD of the brake subsystem is that the data of BC 3-2 is in CF, and its HD is merely 0.2502, which is very close to the fault. When the monitored values of BC 4-1 changes from CF to FS, the HD of the braking subsystem and braking system decrease rapidly, and the HD of the subsystem in which the fault is located is lower than that of the system, which accords with the actual situation and is also helpful to find and locate faults. The above analysis shows the reliability and sensitivity of the method proposed in this paper to some extent.

## 7. Conclusions

According to the actual situation of a mine hoist braking system, we propose a method for calculating the HD of the braking system of a mine hoist combined with TLFCA and BPNN. A method of data standardization is proposed and a unified health membership function is defined, which simplifies the calculation of data health membership. In order to ensure the objectivity, impartiality and scientific rigour of the weight coefficient, this paper puts forward a method of weight determination which combines expert scoring, objective weight determination and AHP. A neural network is used to quantify the HD from FCAVs, which provides technical support for the equipment maintenance decision and subsequent health prediction. The practicability, reliability and sensitivity of the proposed method are proved by the monitored data of the test bed.

The limitation of the method in this paper is that the monitored parameters must reflect the performance of the equipment completely, so obtaining perfect and accurate monitored data is the precondition of using this method. When the data are standardized in this paper, the selection of each data limit should not only be in accordance with the relevant regulations, but also need the knowledge of experts in related fields. The method in this paper requires high computing speed, and needs to consider the configuration of the computer hardware and parallel operation, etc.

After the HD calculation is completed, the HD radar chart of each subsystem and system can be drawn according to the HD of the sensor and subsystem, and the HD trend chart of each sensor, subsystem and system can be drawn. The visual HD chart provides the basis for operation, maintenance and management.

The method proposed in this paper provides a technical support for intelligent maintenance of a braking system such as the residual life prediction and maintenance decision, and provides the necessary data for perfecting the PHM of the braking system. The method can also provide reference for the HD calculation of the whole hoist and other complex equipment.

## Figures and Tables

**Table 1:** The values of mean and standard deviation.

	HC			
	HS	SH	CF	FS
μ	1	0.6	0.3	0
δ	0.2	0.15	0.15	0.15

**Table 2:** Main parameters of test bed.

Name		Specifications
drum	diameter	800 mm
	width	160 mm
hoisting height		47 m
diameter of wire rope		10 mm
payload		1t
volume weight		1t
hoisting speed		1.8 m/s
motor powers		75 × 2 kW
brake number		4 × 2

**Table 3:** The on-line monitored values.

Sensor Name	Data types	Raw data							
		T-1	T-2	T-3	T-4	T-5	T-6	T-7	T-8
BC 1-1	Interval	0.55	0.56	0.45	0.4	0.45	0.5	0.52	0.58
BC 1-2	Interval	1.55	1.56	1.45	1.4	1.45	1.5	1.52	1.58
BC 2-1	Interval	0.885	0.85	0.45	0.6	0.66	0.85	0.8	0.78
BC 2-2	Interval	0.65	0.86	0.45	0.6	0.85	0.65	0.75	0.58
BC 3-1	Interval	1.35	1.16	1.25	0.9	1.55	1.25	1.12	1.08
BC 3-2	Interval	0.05	0.16	0.05	0.1	0.01	0.05	0.052	0.03
BC 4-1	Interval	2.15	1.96	1.85	2.18	1.95	1.95	2.12	2.18
BC 4-2	Interval	1.55	1.56	1.55	1.5	1.55	1.35	1.62	1.68
Temperature	Interval	20	22	20	21	23	20	20	21
Deflection	Cost	0.3	0.4	0.2	0.1	0.1	0.15	0.2	0.3
Motor Current	Cost	1	1.8	2.2	1.9	1.6	1.65	1.78	1.88
Oil Pressure	Interval	11.1	11.2	11.2	11.3	11.2	11.1	11.1	11.1
Oil Temperature	Interval	20	25	28	30	33	33	32	30
Oil Quantity	Interval	135	130	129	128	126	125	126	125
oil pressure of accumulator	Interval	9.5	9.4	9.4	9.4	9.3	9.3	9.25	9.25
Contamination Level	Cost	1	1.1	1.1	1.1	1.2	1.1	1.2	1.1

**Table 4:** FCAVs, HD and HC of the sensors in brake system.

Sensor Name	Fuzzy Comprehensive Assessment Value				HD	HC
	HS	SH	CF	FS		
BC 1-1	0.3216	0.79	0.0493	0.0001	0.7219	SH
BC 1-2	0.3136	0.8072	0.0479	0.0001	0.7183	SH
BC 2-1	0.5914	0.4793	0.0247	0	0.8156	HS
BC 2-2	0.5819	0.4748	0.0193	0	0.8146	HS
BC 3-1	0.6313	0.4471	0.0266	0	0.8259	HS
BC 3-2	0.0015	0.091	0.9293	0.2384	0.2502	CF
BC 4-1	0.0008	0.0462	0.5412	0.6302	0.1371	FS
BC 4-2	0.2173	0.8818	0.1214	0.0005	0.668	SH
Temperature	0.9722	0.0278	0.0000	0.0000	0.9844	HS
Deflection	0.1428	0.4955	0.3555	0.0062	0.5605	SH
Motor Current	0.5554	0.4436	0.0010	0.0000	0.8203	HS
Oil Pressure	0.2571	0.6463	0.0965	0.0001	0.7114	SH
Oil Temperature	0.7415	0.2581	0.0005	0.0000	0.8645	HS
Oil Quantity	0.7415	0.2581	0.0005	0.0000	0.8645	HS
oil pressure of accumulator	0.8089	0.1911	0.0001	0.0000	0.8878	HS
Contamination Level	0.8445	0.1555	0.0000	0.0000	0.9044	HS

**Table 5:** FCAVs, HD and HC of subsystems and brake system.

Name		Fuzzy Comprehensive Assessment Value				HD	HC
		HS	SH	CF	FS	HD	
**Subsystem**	Brake	0.0008	0.0462	0.5412	0.6302	0.1371	FS
	Brake Disc	0.5720	0.2643	0.1794	0.0031	0.8145	HS
	Hydraulic System	0.6785	0.3378	0.0171	0.0000	0.8386	HS
**Brake System**		0.0008	0.0462	0.5412	0.6302	0.1371	FS

**Table 6:** Different BC4-1 monitored values and HD HC of sensor, subsystem or system.

Serial Number	T-1	T-2	T-3	T-4	T-5	T-6	T-7	T-8	BC 4-1		Brake Subsystem		Braking System	
									HD	HC	HD	HC	HD	HC
1	1.08	0.82	0.98	1.23	0.92	1.25	1.13	1.11	0.9803	HS	0.451	CF	0.6944	SH
2	1.55	1.65	1.58	1.63	1.52	1.48	1.55	1.68	0.637	SH	0.3889	CF	0.638	SH
3	1.95	1.96	1.85	1.98	1.95	1.95	1.92	1.88	0.2658	CF	0.3025	CF	0.571	SH
4	2.15	1.96	1.85	2.18	1.95	1.95	2.12	2.18	0.1371	FS	0.1371	FS	0.1371	FS

## Nomenclature

HD	health degree
PHM	Prognostics Health Management
CBM	condition based maintenance
TLFCA	three-level fuzzy comprehensive assessment
BPNN	BP neural network
FCA	fuzzy comprehensive assessment
FCAV	fuzzy comprehensive assessment value
AHP	analytic hierarchy process
HS	health state
SH	sub-health
CF	critical fault
FS	fault state
BC	brake clearance
HC	health condition
$X_{i}$	the monitored values of the *i*th sensor, $X_{i}=\left[x_{1}^{i},x_{2}^{i},\cdots ,x_{k}^{i}\right]$
$HD_{SENSOR}$	HD of sensor
$HD_{SUB-SYS}$	HD of subsystem
$HD_{SYS}$	HD of system
$FCAV_{SENSOR}$	FCAVs of sensor
$FCAV_{SUB-SYS}$	FCAVs of subsystem
$FCAV_{SYS}$	FCAVs of system
U	factor sets, $U=\left[U_{1},U_{2},\cdots ,U_{S}\right]$
$U_{i}$	factor classes, i=1,2,…,s
$U_{ij}$	the *j*th factor in *i*th factor class.
V	assessment sets, $V=\left[V_{1},V_{2},\cdots ,V_{m}\right]$
W	weight sets of the factor sets, $W=\left[W_{1},W_{2},\cdots ,W_{s}\right]$
$\omega_{i}$	weight sets of the *i*th factor class or subsystem, $\omega_{i}=\left[\omega_{i1},\omega_{i2},\cdots ,\omega_{ik_{i}}\right]$
$w_{i}$	weight sets of the *i*th sensor, $w_{i}=\left[w_{i1},w_{i2},\cdots ,w_{ik}\right]$.
$w_{i}^{1}$	weight sets of time point obtained by experts scoring method.
$w_{i}^{2}$	weight sets of the *i*th sensor obtained by hierarchy method
R	transfer matrix of factor sets and assessment sets
$R_{i}$	transfer matrix of the *i*th factor class or subsystem and assessment sets
$r_{i}$	transfer matrix of the *i*th sensor
$R_{jl}^{i}$	fuzzy conversion value between the *j*th factor in the *i*th factor class and the *l*th assessment subset
FCAV	FCAVs of factor sets
$FCAV_{i}$	FCAVs of the *i*th factor class, sensor or subsystem.
$FCAV^{l}$	FCAV of the factor sets belongs to the *l*th assessment factor.
$FCAV_{il}$	FCAV of the *l*th assessment factor in the *i*th factor class
x	the monitored value of the sensor.
$x_{\min1}$	the lower limit value of the lowest operation
$x_{\min2}$	the lower limit value of the best operation
$x_{\max2}$	the upper limit value of the best operation
$x_{\max1}$	the upper limit value of the lowest operation
$f_{V_{l}}\left(x\right)$	membership function of assessment sets
$V_{l}$	different HC, l=1,2,3,4.
$\delta_{l}$	standard deviation of the *l*th HC
$\mu_{l}$	the optimal expected value of the *l*th HC.
$d_{ij}$	the scale value of the *i*th parameter at *j*th point.
$c_{ij}$	the absolute value of the difference between the monitored and optimal expected value of the *i*th parameter at *j*th point.
$s_{ij}$	the absolute value of the difference between the optimal expectation of the health and fault of the *i*th parameter.
$F_{HSn}^{i}$	FCAV of the *i*th sensor at *n*th point is in HS
$F_{SHn}^{i}$	FCAV of the *i*th sensor at *n*th point is in SH
$F_{CFn}^{i}$	FCAV of the *i*th sensor at *n*th point is in CF
$F_{FSn}^{i}$	FCAV of the *i*th sensor at *n*th point is in FS
$FCAV_{HS}^{i}$	FCAV of the *i*th sensor or *i*th subsystem is in HS
$FCAV_{SH}^{i}$	FCAV of the *i*th sensor or *i*th subsystem is in SH
$FCAV_{CF}^{i}$	FCAV of the *i*th sensor or *i*th subsystem is in CF
$FCAV_{FS}^{i}$	FCAV of the *i*th sensor or *i*th subsystem is in FS
